# 2420 Examining characteristics of placebo effects on trauma-related insomnia in a suvorexant trial

**DOI:** 10.1017/cts.2018.167

**Published:** 2018-11-21

**Authors:** Kimberly Uweh

**Affiliations:** Georgetown – Howard Universities

## Abstract

OBJECTIVES/SPECIFIC AIMS: The aims of this project are to: (1) examine placebo effects on subjective and objective outcome measures, (2) determine if an increase in the placebo is associated with changes in benefit, (3) evaluate if the trauma related insomnia placebo group in our study has different side effect reports compared with insomnia placebo participants in previous suvorexant trials, and (4) (Exploratory) examine associations between the placebo group’s characteristics (e.g., trauma/PTSD severity, demographics) and placebo effects. METHODS/STUDY POPULATION: The parent study is a randomized double-blind placebo-controlled clinical trial (clinicaltrials.gov ID: NCT02704754) of suvorexant for treatment of adults (age 18–55) with insomnia that started or worsened after trauma exposure. Suvorexant is a first in class orexin antagonist and is approved by the FDA for the indication of insomnia. In this 6-week trial, all participants initially take 10 mg of suvorexant/placebo, and the dose will be increased to 20 mg if participants continue to experience clinically significant insomnia symptoms 1 week after starting the medication. Sleep outcomes will be measured by polysomnography, daily sleep diary, and the Insomnia Severity Index. RESULTS/ANTICIPATED RESULTS: We hypothesize that (1) within the placebo group data both subjectively and objectively measured outcomes will similarly show improvement in insomnia symptoms, (2) the increase of the placebo medication dose will result in an increased benefit, (3) the trauma-related insomnia placebo group will have the same type and similar rate of side effects reported in previous suvorexant trials. DISCUSSION/SIGNIFICANCE OF IMPACT: Most previous studies examining placebo effects focused on pain and depression. Information obtained from this project will complement our current understanding of placebo effects by characterizing placebo effects on trauma-related insomnia. This study will inform the development of novel strategies to maximize utility of placebos in future clinical trials.

